# *Caenorhabditis elegans* provides an efficient drug screening platform for *GNAO1*-related disorders and highlights the potential role of caffeine in controlling dyskinesia

**DOI:** 10.1093/hmg/ddab296

**Published:** 2021-10-08

**Authors:** Martina Di Rocco, Serena Galosi, Enrico Lanza, Federica Tosato, Davide Caprini, Viola Folli, Jennifer Friedman, Gianfranco Bocchinfuso, Alberto Martire, Elia Di Schiavi, Vincenzo Leuzzi, Simone Martinelli

**Affiliations:** Department of Oncology and Molecular Medicine, Istituto Superiore di Sanità, Rome 00161, Italy; Department of Human Neuroscience, ‘Sapienza’ University of Rome, Rome 00185, Italy; Department of Human Neuroscience, ‘Sapienza’ University of Rome, Rome 00185, Italy; Center for Life Nano Science, Istituto Italiano di Tecnologia, Rome 00161, Italy; Department of Oncology and Molecular Medicine, Istituto Superiore di Sanità, Rome 00161, Italy; Center for Life Nano Science, Istituto Italiano di Tecnologia, Rome 00161, Italy; Center for Life Nano Science, Istituto Italiano di Tecnologia, Rome 00161, Italy; Department of Neuroscience and Department of Pediatrics, University of California, San Diego, CA 92123, USA; Division of Neurology, Rady Children’s Hospital San Diego, CA 92123, USA; Rady Children’s Institute for Genomic Medicine, San Diego, CA 92123, USA; Department of Chemical Sciences and Technologies, University of Rome ‘Tor Vergata’, Rome 00133, Italy; National Center for Drug Research and Evaluation, Istituto Superiore di Sanità, Rome 00161, Italy; Institute of Biosciences and BioResources, National Research Council, Naples 80131, Italy; Department of Human Neuroscience, ‘Sapienza’ University of Rome, Rome 00185, Italy; Department of Oncology and Molecular Medicine, Istituto Superiore di Sanità, Rome 00161, Italy

## Abstract

Dominant *GNAO1* mutations cause an emerging group of childhood-onset neurological disorders characterized by developmental delay, intellectual disability, movement disorders, drug-resistant seizures and neurological deterioration. *GNAO1* encodes the α-subunit of an inhibitory GTP/GDP-binding protein regulating ion channel activity and neurotransmitter release. The pathogenic mechanisms underlying *GNAO1*-related disorders remain largely elusive and there are no effective therapies. Here, we assessed the functional impact of two disease-causing variants associated with distinct clinical features, c.139A > G (p.S47G) and c.662C > A (p.A221D), using *Caenorhabditis elegans* as a model organism. The c.139A > G change was introduced into the orthologous position of the *C. elegans* gene via CRISPR/Cas9, whereas a knock-in strain carrying the p.A221D variant was already available. Like null mutants, homozygous knock-in animals showed increased egg laying and were hypersensitive to aldicarb, an inhibitor of acetylcholinesterase, suggesting excessive neurotransmitter release by different classes of motor neurons. Automated analysis of *C. elegans* locomotion indicated that *goa-1* mutants move faster than control animals, with more frequent body bends and a higher reversal rate and display uncoordinated locomotion. Phenotypic profiling of heterozygous animals revealed a strong hypomorphic effect of both variants, with a partial dominant-negative activity for the p.A221D allele. Finally, caffeine was shown to rescue aberrant motor function in *C. elegans* harboring the *goa-1* variants; this effect is mainly exerted through adenosine receptor antagonism. Overall, our findings establish a suitable platform for drug discovery, which may assist in accelerating the development of new therapies for this devastating condition, and highlight the potential role of caffeine in controlling *GNAO1*-related dyskinesia.

## Introduction

Dominant mutations in the *GNAO1* gene (RefSeq NG_042800.1, NM_020988.3) underlie a complex constellation of infantile/childhood-onset severe neurological disorders characterized by global developmental delay (GDD), intellectual disability (ID), hypotonia, movement disorders (MD), drug-resistant seizures and neurological deterioration (1,2). Originally, *de novo* variants were associated with early infantile epileptic encephalopathy [EIEE17, MIM #615 473; (3)]. More recently, the phenotypic spectrum of this condition has been expanded by the identification of allelic changes causing a neurodevelopmental disease with hypotonia and MD as prominent features [NEDIM, MIM #617 493; (4–7)]. Besides these traits, many patients present with various combinations of MD, epilepsy and GDD. MD is usually early and severe, with life-threatening paroxysmal dyskinetic status induced by recognizable triggers including infections, fever and emotions. These precipitating factors influence the clinical evolution of this condition and contribute to neurological decline and lethality with mechanisms still to be explored (8).

*GNAO1* encodes the α-subunit (Gα) of a heterotrimeric guanine nucleotide-binding protein (G_o_), which is highly expressed in the mammalian brain, particularly in cerebral cortex, hippocampus and striatum (9). G_o_ modulates inhibitory signaling from G-protein coupled receptors (GPCRs), including GABA_B_, dopamine D_2_, α_2A_ adrenergic and adenosine A_1_, regulating neuronal excitability and neurotransmission (10), and controlling neurodevelopment (11). In the brain, G_o_ negatively controls the production of cyclic adenosine monophosphate (cAMP) by inhibiting adenylyl cyclase (AC), directly prevents neurotransmitter release, inhibits N-type and P/Q-type calcium channels and activates G protein-coupled inward rectifying potassium (GIRK) channels. Gα_o_ is part of a complex network of proteins regulating intracellular cAMP levels involved in the pathophysiology of hyperkinetic MD [e.g. ADCY5, GNB1, GNAL1, GPR88 and PDE10E; (12)]. Accordingly, aberrant cAMP synthesis has been proposed as the pathogenic mechanism of the disease, with loss-of-function (LOF) and gain-of-function (GOF) alleles that appeared to be primarily associated with epilepsy and MD, respectively (13). These data apparently contradict the original findings from Nakamura *et al.* (3) suggesting a LOF behavior of *GNAO1* variants on Gα_o_-mediated signaling, regardless of the associated clinical presentation. More recently, *GNAO1* variants were shown to disturb Gα_o_ function in a cell-type-specific manner via a combination of LOF and dominant-negative (DN) mechanisms that are not mutually exclusive (14). Based on these data, several questions regarding the pathogenic mechanisms and the genotype/phenotype correlations remain unanswered. This lack of knowledge hinders the development of effective therapies. To date, the spectrum of pharmacological options for *GNAO1*-related disorders is extremely narrow. Tetrabenazine, the most effective drug in the baseline management of dyskinesia, is ineffective in controlling MD exacerbations and deep brain stimulation (DBS) becomes necessary in the most severe hyperkinetic forms (15,16). Similarly, seizures in EIEE17 can be refractory to combinations of multiple anti-epileptic agents (2).

Human disease-causing genes often function in evolutionarily conserved pathways, which can be dissected in simple model organisms. Given the simplicity and well-described anatomy of its nervous system, and the similarity with vertebrate neuronal pathways, the nematode *Caenorhabditis elegans* has emerged as a powerful tool for modeling human diseases, particularly neurological disorders (17). For multiple conditions, this invertebrate has enabled new strategies for therapeutic interventions and provided a whole-animal setting for genetic and chemical screening. Similar to its mammalian counterpart, nematode Gα_o_ is highly expressed in the nervous system and some muscle cells (18,19). In *C. elegans* neurons, Gα_o_ signaling inhibits the neurotransmitter release machinery (reviewed in 20). Specifically, Gα_o_ prevents acetylcholine release by ventral cord motor neurons, inhibiting locomotion (21–24) and controls the release of serotonin and other neurotransmitters by HSNs, a pair of motor neurons that innervate vulval muscles in the hermaphrodite, inhibiting egg laying (25). Accordingly, Gα_o_ null mutants show hyperlocomotion and increased egg laying (18,19).

Here, we assessed the functional consequences of two *GNAO1* pathogenic variants *in vivo* using *C. elegans* as a model system. The first variant, c.139A > G (p.S47G), occurring in a boy with a severe phenotype including both epileptic encephalopathy and MD, was introduced at the orthologous position of the worm gene using CRISPR/Cas9. A knock-in strain carrying a second variant, c.662C > A (p.A221D), which is associated with clinical features resembling NEDIM, was already available at the *Caenorhabditis* Genetic Center (CGC). Functional studies revealed a strong hypomorphic effect of both variants on Gα_o_-mediated signaling, with a partial DN activity limited to the p.A221D allele. Of note, caffeine was found to significantly improve aberrant motor function of mutant animals by blocking a putative adenosine receptor (AR) in the nematode. Our findings establish an experimental platform for drug discovery and highlight the potential role of caffeine and other AR antagonists in controlling *GNAO1*-related hyperkinetic MD.

## Results

### Clinical findings

The 10-year-old boy harboring the c.139A > G (p.S47G) variant was previously reported with limited clinical information in Danti *et al*. (8). Here, we report his follow-up with emphasis on the attempted therapeutic strategies. He was born at term after a normal pregnancy and delivery from non-consanguineous healthy parents. Family history was negative for genetic or neurological diseases. At birth, Apgar score was 7/9, weight 3130 g (32° centile), height 51 cm (70° centile) and occipital frontal circumference 34 cm (36° centile). Breastfeeding difficulties and weakness were noticed in the first months of life followed by progressive developmental delay with hypotonia, dystonic movements of limbs and excessive startle reflex, from the fifth month of life. At age 18–20 months, he was able to inconsistently control his head, to roll over, to transfer objects in his hands and to babble, but a few months later he experienced gradual neurological deterioration associated with a marked increase of MD. At the age of 27 months, he exhibited GDD with lack of postural reactions, severe hypotonia and hypokinesia, and dystonic posture and movements intermingled with occasional spells of generalized choreic and ballistic dyskinesias, triggered by voluntary attempt to move, loss of postural control, startle reflexes or emotional stress. Language was absent while social interaction and eye contact were preserved. An extensive neurometabolic work-up was unremarkable, apart from a slight reduction of 5-methyltetrahydrofolate in CSF (34 nmol/l; n.v., 63–111) with normal biogenic amines. Brain magnetic resonance imaging (MRI) and ^1^H-MR spectroscopy performed at 10 and 26 months of age were unremarkable. Serial video EEG recordings showed rolandic paroxysmal abnormalities not associated with epileptic seizures.

Tetrabenazine administration (1.5 mg/kg/day progressively titrated to 3 mg/kg/day) from age 29 months persistently improved spontaneous but not evoked paroxysmal dyskinesias. Mood swings, bruxism and a tendency of pulling his hair, lip biting and hand-washing stereotypies were intermittently observed. From the age of 3 years, he suffered from focal epileptic seizures with secondary generalization, mainly emerging before and during sleep, which could be partially controlled by Topiramate (4 mg/kg/day) and then Carbamazepine (20 mg/kg/day). In the following years, the clinical course was characterized by a rising frequency of paroxysmal life-threatening dyskinetic status lasting few minutes to several hours, triggered by environmental or physical events, such as fever, seasonal variations in temperature and emotional stress, which left the patient exhausted and debilitated. Addition of clonidine (3.75 mg/kg/day) and lorazepam (2 mg/day) was minimally effective in controlling motor exacerbations. Eventually, at the age of 10 years, he underwent surgical implantation of globus pallidus internus DBS device, which demonstrated effective in controlling major paroxysmal motor episodes. Brain MRI at age 10 revealed enlargement of frontal and temporal spaces interpreted as incipient cortical atrophy.

The c.662C > A (p.A221D) variant was reported as a *de novo* mutation in two unrelated individuals with clinical features within the phenotypic spectrum of *GNAO1*-related disorders (ClinVar: #SCV000924543.1 and #SCV000746624.2). A first subject showed GDD, dystonia, parkinsonism, mild ID, motor and speech delay, hypotonia and EEG abnormalities, resembling features occurring in NEDIM (26). No clinical details are reported for the second patient.

### Structural analysis

Similar to the α-chain of other heterotrimeric guanine nucleotide-binding proteins, Gα_o_ consists of two domains: a Ras-like GTPase domain and an α-helical domain (27). Both affected residues, Ser^47^ and Ala^221^, belong to the Ras-like GTPase domain (Supplementary Material, Fig. S1A). In particular, Ser^47^ is located close to the interface between the two domains and is part of the P-loop motif (Supplementary Material, Fig. S2), which is directly involved in the interaction with the nucleotide and the Mg^2+^ cation. This loop plays a key role in the allosteric regulation of the protein, since its structure changes during the activation and deactivation steps (28). Therefore, amino acid changes involving residues located within this motif are predicted to perturb Gα function. More specifically, Ser^47^ interacts with Mg^2+^ in the GTP-bound structure (Supplementary Material, Fig. S1B), and the serine-to-glycine substitution is expected to perturb this interaction. Since the Mg^2+^ cofactor increases the affinity for GTP of three orders of magnitude (29), mutations affecting the P-loop motif, including p.S47G, are defective in GTP binding (14).

Ala^221^ is part of a β4 motif in an extended β-sheet region located in the core of the Ras-like GTPase domain, close to switch II [residues 200–220; (30); Supplementary Material, Fig. S2]. The side chain of Ala^221^ is part of a hydrophobic core with residues Leu^36^, Ile^266^, Val^339^ and Ile^342^ (Supplementary Material, Fig. S1C). Among these, Val^339^ and Ile^342^ are located in the α5 helix, which is determinant in the interaction between the α-chain and the cognate GPCRs, including the serotonin 5-HT1B receptor reported in Supplementary Material, Fig. S1 (31). By introducing a net charge in the hydrophobic pocket, the p.A221D substitution is predicted to perturb this key structural motif and, in turn, proper GPCR-binding. Because of the close proximity of Ala^221^ to the switch II region, however, an effect on the dynamical features of this region cannot be ruled out.

### Generation and phenotypic characterization of a GNAO1 *C. elegans* model using CRISPR/Cas9

*goa-1*, the *C. elegans* ortholog of *GNAO1*, shows 90% homology with the human gene, with > 80% identity in amino acid sequence (www.ncbi.nlm.nih.gov/homologene; Supplementary Material, Fig. S2). Here, we introduced the c.139_141TCG > GGA (p.S47G) nucleotide change at the orthologous position of the *C. elegans* gene by CRISPR/Cas9 to generate *goa-1*(*pan5*[S47G]) animals (hereafter *goa-1*[S47G]). The resulting nematodes, homozygous for the desired variant, showed ‘slow growth’ and a ‘protruding vulva’ (Pvl) phenotype (15% in adult hermaphrodites; *n* = 200), resembling features reported following RNAi-mediated knockdown of *goa-1* (32) as well as those observed in knock-out worms carrying the *goa-1*(*sa734*) null allele [c.154C > T; p.Q52^*^; (33,34)]. Similar to null mutants, knock-in lines exhibited low penetrant embryonic lethality and larval arrest (Supplementary Material, Fig. S3). These generalized phenotypes were also observed in *goa-1*(*knu751*) animals carrying the c.662C > A (p.A221D) variant (hereafter *goa-1*[A221D]). This strain was available at the CGC (https://cgc.umn.edu/strain/COP1863); mutant animals were described as hyperactive and show a Pvl phenotype.

**Figure 1:**
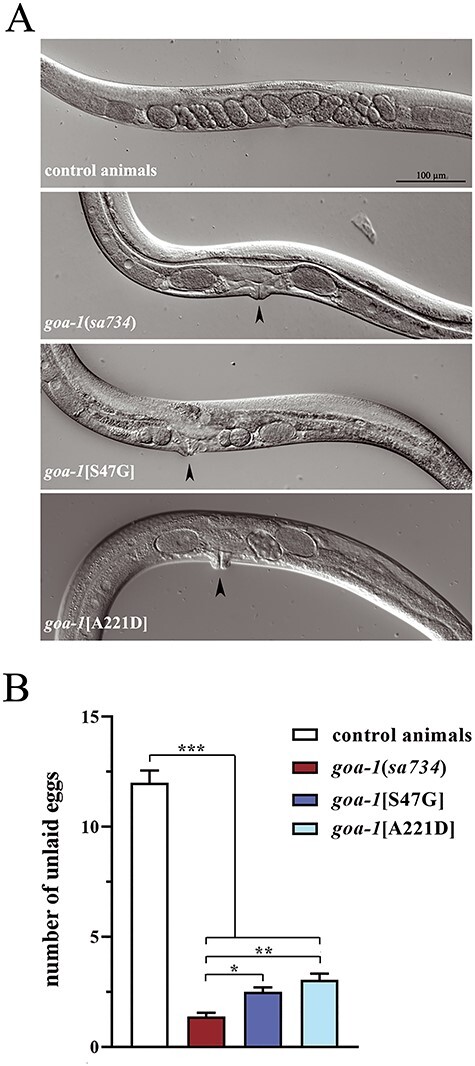
*goa-1*[S47G] and *goa-1*[A221D] animals display increased egg laying activity. (**A**) Representative images of adult mid-body regions of wild-type *C. elegans* (upper panel) and *goa-1* mutants (lower panels). Unlaid eggs are evident as oval objects inside the body. Control animals display 12–16 unlaid eggs on average, whereas homozygous Gα_o_ mutants retain only 1–3 eggs. A protruding vulva phenotype is also visible in mutant animals (black arrows). Magnification is the same in all images. (**B**) The increased egg laying activity is quantified as number of eggs retained in the uterus of adult hermaphrodites (^*^P < 0.05, ^*^^*^P < 0.001 and ^*^^*^^*^P < 0.0001; one-way ANOVA with Bonferroni correction). Twenty animals for each genotype were tested. Data represent means ± SEM of multiple observations.

**Figure 2:**
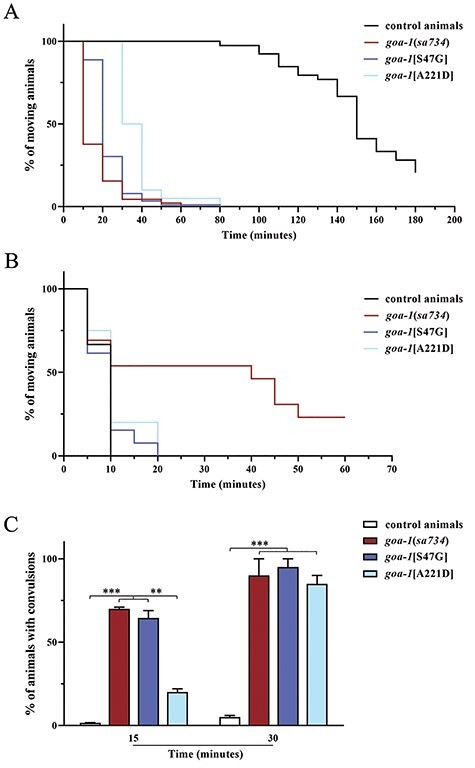
*goa-1*[S47G] and *goa-1*[A221D] animals show increased acetylcholine release at the *C. elegans* NMJ. (**A**) Homozygous Gα_o_ mutants are hypersensitive to aldicarb-induced paralysis (1 mm) (*P* < 0.0001 in all comparisons; log-rank test), suggesting excessive acetylcholine release at the NMJ. Knock-in animals display a milder phenotype compared with null mutants (*goa-1*[S47G], *P* < 0.02; *goa-1*[A221D], *P* < 0.0001). *goa-1*[A221D] animals show a milder phenotype compared with *goa-1*[S47G] (*P* < 0.0001). Number of animals tested: control, *n* = 39; *goa-1*(*sa734*), *n* = 45; *goa-1*[S47G], *n* = 90; *goa-1*[A221D], *n* = 20. (**B**) *goa-1*[S47G] and *goa-1*[A221D] animals show normal sensitivity to the paralyzing effect of the cholinergic agonist levamisole (1 mm), supporting a presynaptic origin of the defect, whereas *goa-1* null mutants display a slight resistance to the drug (*P* < 0.05). Twenty animals for each genotype were tested. (**C**) Gα_o_ mutants display hypersensitivity to PTZ-induced convulsions (5 mg/ml on agar plates), likely indicating defective GABA versus acetylcholine release by ventral cord motor neurons (^*^^*^P < 0.001; ^*^^*^^*^P < 0.0001 in all comparisons; Fisher’s exact test with Bonferroni correction). Twenty animals for each genotype were tested. Data represent means ± SEM of three independent experiments.

To explore the impact of the p.S47G and p.A221D amino acid changes on Gα_o_ function and to distinguish between GOF and LOF effect, we counted the number of eggs retained in the uterus of gravid hermaphrodites and assessed acetylcholine release at the neuromuscular junction (NMJ). Unlaid eggs are easily visible inside the body of adult animals (Fig. 1A). Wild-type hermaphrodites normally display 12–16 unlaid eggs on average. In contrast, homozygous null mutants and *goa-1*[S47G] and *goa-1*[A221D] knock-in nematodes laid almost all their eggs as soon as they are generated, accumulating only 1–3 eggs in their uterus (Fig. 1A and B), suggesting defective Gα_o_ function in HSN neurons. Mutant animals also displayed a reduced brood size compared with control worms that, however, cannot explain *per se* the extremely low number of retained eggs (Supplementary Material, Fig. S3). Given the established role of Gα_o_ signaling in the negative control of acetylcholine release from ventral cord motor neurons onto body-wall muscles (21–24), LOF/hypomorphic mutations affecting *goa-1* or its signaling pathway were shown to alter the response to aldicarb, an inhibitor of acetylcholinesterase, an enzyme that clears acetylcholine from the synaptic cleft (35). In control nematodes, exposure to aldicarb results in sustained muscle contraction and paralysis. Of note, homozygous *goa-1*[p.S47G] and *goa-1*[A221D] animals behave as null mutants displaying hypersensitivity to aldicarb-induced paralysis, suggesting excessive release of acetylcholine at the NMJ (Fig. 2A). Knock-in animals showed normal sensitivity to levamisole, a strong nicotinic agonist acting on postsynaptic muscles to cause hypercontraction and paralysis, further supporting the presynaptic origin of the cholinergic defect (Fig. 2B). It is worth noting, however, that *goa-1* null mutants displayed a slight resistance to levamisole, suggesting a possible compensatory post-synaptic effect that needs to be further investigated. Finally, we assessed the relative extent of GABAergic signaling by treating worms with pentylenetetrazole (PTZ). In *C. elegans*, body-wall muscles receive signals from both excitatory (cholinergic) and inhibitory (GABAergic) motor neurons to generate an alternating wave of contraction and relaxation, allowing proper locomotion (36–38). PTZ is a competitive inhibitor of GABA, preventing its binding to GABA_A_ receptors on body-wall muscles. Exposure to PTZ leads to a shift in the equilibrium between excitatory and inhibitory inputs towards the former, resulting in a convulsion phenotype. All mutants displayed hypersensitivity to PTZ (Fig. 2C and Supplementary Material, Fig. S4), indicating defective GABAergic signaling, which is likely because of augmented release of acetylcholine at the *C. elegans* NMJ. Overall, these findings demonstrate defective Gα_o_-mediated inhibition of neurotransmitter release by HSN and ventral cord motor neurons in both *goa-1*[S47G] and *goa-1*[A221D] animals. Of note, *goa-1*(*sa734*) null mutants showed a more penetrant phenotype compared with both knock-in animals, pointing to a strong hypomorphic rather than a complete LOF effect of the p.S47G and p.A221D disease-causing variants.

**Figure 3:**
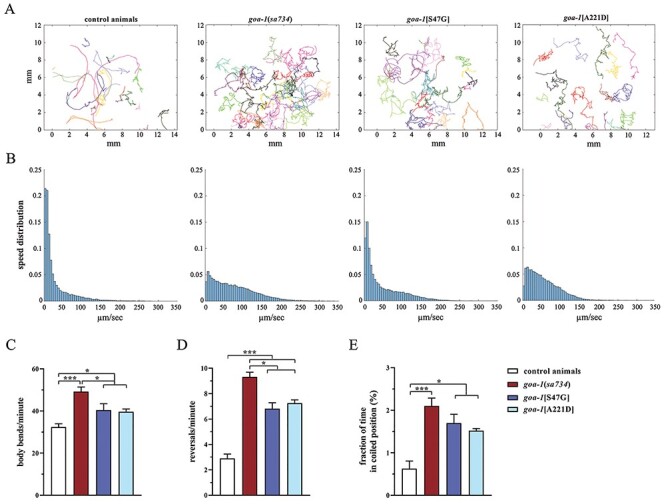
*goa-1*[S47G] and *goa-1*[A221D] animals exhibit aberrant locomotion. (**A**) Trajectories of multiple *C. elegans* (*n* = 20) on 35 mm plates seeded with a thin lawn of *E.coli* OP50 bacteria. Animals were recorded for 10 min. Different colors refer to different nematodes. All mutants show hyperactive crawling, with a higher frequency of change in direction, compared with controls. (**B**) Speed histograms of the same tracks indicate a clear difference between wild-type and mutant animals, the latter being faster on average (means are 29.3, 75.5, 47.9 and 55.8 μm/s for N2, *goa-1*(*sa734*), *goa-1*[p.S47G] and *goa-1*[A221D], respectively; *P* < 10^−6^, two-sample *t*-test). The aberrant locomotor activity of *goa-1* mutants is also revealed by the higher number of body bends per minute (^*^P < 0.02 and ^*^^*^^*^P < 0.0001; one-way ANOVA with Bonferroni correction) (**C**) and reversals per minute (^*^P < 0.002; ^*^^*^^*^P < 0.0001) (**D**), compared with control animals and the average fraction of time spent in a coiled position (^*^P < 0.01 and ^*^^*^^*^P < 0.0001) (**E**). Number of animals tested (C–E): controls, *n* = 27; *goa-1*(*sa734*), *n* = 57; *goa-1*[S47G], *n* = 84; *goa-1*[A221D], *n* = 20. Data represent means ± SEM of multiple observations.

In null *goa-1* mutants, increased acetylcholine release from the ventral cord motor neurons leads to hyperactive locomotion (18,24). Here, to characterize the locomotor behavior of knock-in worms, we used a custom automated tracking system (Supplementary Material, Fig. S5). Computational analysis revealed that *goa-1*[S47G] and *goa-1*[A221D] mutants are characterized by hyperactive crawling (Fig. 3A) and tend to move faster than control animals (Fig. 3B). In line with that, they showed more frequent (and deeper) body bends compared with wild-type worms (Fig. 3C). Furthermore, knock-in nematodes exhibited more frequent reversals (change of direction; Fig. 3D) than control animals. Finally, *goa-1*[S47G] and *goa-1*[A221D] mutants displayed uncoordinated locomotion, assuming a tightly coiled posture and tending to remain stationary in this position rather than actively moving on the plate as control nematodes (‘coiler’ phenotype) (Fig. 3E). Again, a more severe phenotype was observed in *goa-1*(*sa734*) null mutants, further supporting the hypomorphic effect of both variants.

**Figure 4:**
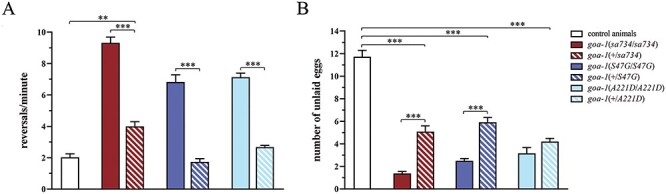
Phenotypic analysis of heterozygous animals reveals that p.S47G and p.A221D have a different behavior. Number of reversals per minute (**A**) and number of unlaid eggs (**B**) were counted in animals carrying the *goa-1* nonsense or missense variants in a heterozygous state (F1 progeny). Wild-type animals were also auto-crossed for comparison. *goa-1(+/sa734)* null heterozygotes display a residual phenotype in terms of both number of unlaid eggs (*P* < 0.0001; two-way ANOVA with Bonferroni correction) and increased reversal rate (*P* < 0.05), although less severe than that observed in homozygous null mutants (*P* < 0.0001), indicating that *goa-1* is a haploinsufficient gene for these particular phenotypes. Heterozygous knock-in mutants show a residual phenotype only in terms of unlaid eggs (*P* < 0.0001), which is milder compared with that of their homozygous counterparts (^*^^*^^*^P < 0.0001 in all comparisons), except for *goa-1(+/A221D)*, indicating that p.A221D has a partial a DN effect on egg laying function. Fifty animals for each genotype were assessed. Data represent means ± SEM of multiple experiments performed using three independent clones obtained following genetic crosses to wild-type animals.

To explore a possible DN effect of p.S47G and p.A221D, we analyzed the F1 progeny after crossing homozygous mutant hermaphrodites to control males carrying the wild-type *goa-1* allele. Our data showed that *goa-1(+/sa734)* null heterozygotes display a clear phenotype in terms of both number of unlaid eggs and increased reversal rate, although less severe than that observed in homozygous null mutants (Fig. 4A and B). This semi-dominant effect indicates that *goa-1* is a haploinsufficient gene for these particular phenotypes. Phenotypic analysis of heterozygous knock-in animals showed that p.S47G and p.A221D behave differently from each other. *goa-1(+/S47G)* nematodes displayed a weaker phenotype compared with heterozygous null mutants, which was similar to wild-type in the number of reversals per minute (Fig. 4A) and similar to *goa-1(+/sa734)* in the number of unlaid eggs (Fig. 4B), indicating that p.S47G has no DN activity. Contrariwise, *goa-1(+/A221D)* mutants exhibited a slightly more severe phenotype compared with heterozygous null mutants in terms of number of unlaid eggs. More importantly, the prevalence of this phenotype was similar to that observed in *goa-1*[A221D] homozygous animals. Specifically, the percentage of rescue in heterozygous *versus* homozygous animals was 35, 36 and 11% in *goa-1(+/sa734)*, *goa-1(+/S47G)* and *goa-1(+/A221D)*, respectively (*P* < 0.001 between *goa-1(+/A221D)* and the other mutants; Student’s *t*-test), suggesting a DN effect of p.A221D on egg laying.

### Caffeine ameliorates aberrant locomotor behavior of goa-1 mutants

**Figure 5:**
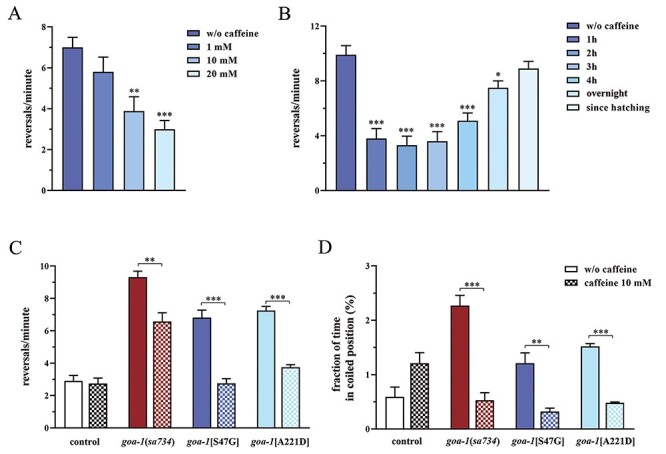
Caffeine suppresses increased reversals and uncoordinated locomotion in *goa-1* mutants. (**A**) Exposure to caffeine (2 h) rescues the increased reversal rate of *goa-1*[S47G] animals in a dose-dependent manner (^*^^*^P = 0.0025 and ^*^^*^^*^P < 0.0001; one-way ANOVA with Bonferroni correction). (**B**) Longer exposures to caffeine (10 mm) is less efficient in ameliorating the reversal phenotype of *goa-1*[S47G] worms compared with acute exposures (^*^P < 0.05 and ^*^^*^^*^P < 0.0001). Twenty animals were assayed for each condition (A, B). Computational analysis reveals that caffeine (10 mm, 2 h) is able to diminish the number of reversals per minute of *goa-1*(*sa734*) null mutants and *goa-1*[A221D] animals, leaving unaffected the reversal rate of control nematodes (^*^^*^P < 0.001 and ^*^^*^^*^P < 0.0001) (**C**) and the time spent by all mutants in a coiled position (^*^^*^P < 0.002 and ^*^^*^^*^P < 0.0001) (**D**). Number of animals tested (C, D): controls, *n* = 27; controls + caffeine, *n* = 61; *goa-1*(*sa734*), *n* = 57; *goa-1*(*sa734*) + caffeine, *n* = 31; *goa-1*[S47G], *n* = 84; *goa-1*[S47G] + caffeine, *n* = 39; *goa-1*[A221D], *n* = 20; *goa-1*[A221D] + caffeine, *n* = 20. Data represent means ± SEM of four independent experiments.

The aforementioned phenotypes are very easy to score under a stereomicroscope, thus representing excellent phenotypic outputs to test the ability of candidate molecules to restore or compensate proper Gα_o_ function. Based on our findings, *C. elegans* has emerged as a suitable platform for drug screening or validation in *GNAO1*-related disorders. As a proof-of-concept, we assessed the effect of caffeine on abnormal locomotion of *goa-1* mutant animals. Caffeine is a psychotropic agent acting as an antagonist of adenosine GPCRs in mammals (39). Recent observations suggest that this drug may improve motor symptoms in subjects with dominant mutations in the *ADCY5* gene (40,41), coding for the cAMP-generating enzyme adenylyl cyclase 5 (AC5), the predominant AC isoform in the striatum (42). GOF variants in this gene underlie familial dyskinesia with facial myokymia (MIM # 606 703), an autosomal dominant childhood-onset disease characterized by hyperkinetic MD resembling that observed in NEDIM (12). Here, exposure of *goa-1*[S47G] animals to caffeine (2 h) was shown to rescue aberrant locomotion in terms of number of reversals per minute in a dose-dependent manner (Fig. 5A). Of note, longer or ‘chronic’ exposure to the drug appeared to be less efficient in improving the reversal frequency compared with shorter, ‘acute’ exposures (Fig. 5B). For these experiments, the lowest effective mm concentration has been used since 10 mm caffeine already proved to protect dopaminergic neurons from dopamine-induced neurodegeneration (43) and to reduce aberrant locomotion induced by L-DOPA in a transgenic *C. elegans* model of Parkinson’s disease [PD; (44)]. Automated recordings of *C. elegans* trajectories highlighted the beneficial effect of caffeine (10 mm) on the hyperactive crawling of *goa-1*[S47G] and *goa-1*[A221D] mutants as well as of animals lacking *goa-1* (Supplementary Material, Fig. S6). Computational analysis revealed that caffeine is able to reduce the number of reversals per minute of knock-in and knock-out worms, whereas the reversal rate was not affected in control animals (Fig. 5C). Furthermore, the time spent by *goa-1* mutants in a coiled position decreased following exposure to the drug, whereas an opposite effect was noted in wild-type nematodes (Fig. 5D). These findings demonstrate that caffeine acts as a bypass suppressor, as it suppresses aberrant motor function even in null mutants, thus bypassing the requirement for the *goa-1* gene.

**Figure 6:**
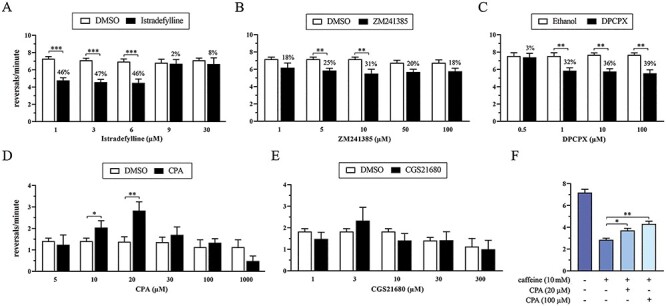
The beneficial effect of caffeine on the increased reversal rate is exerted, in part, through AR antagonism. Caffeine-induced reduction of the reversal rate observed in *goa-1*[S47G] animals is mimicked by exposure to the selective A_2A_R antagonists istradefylline (**A**) and ZM241385 (**B**), as well as to the selective A_1_R antagonist DPCPX (**C**) (^*^^*^P < 0.005 and ^*^^*^^*^P < 0.0001; two-way ANOVA with Bonferroni correction). Results are also given as percentage of rescue of the abnormal reversal rate, which is defined as described in Materials and Methods. Contrariwise, the number of reversals per minute is increased in wild-type nematodes exposed to the selective A_1_R agonist CPA (^*^P < 0.05 and ^*^^*^P < 0.005) (**D**), whereas no effect is visible following administration of the A_2A_R agonist CGS21680 (**E**). Worth noting, when challenged against caffeine, CPA is able to counteract the positive effect of caffeine by increasing the reversal rate of *goa-1*[S47G] mutants (^*^P < 0.05 and ^*^^*^P < 0.005) (**F**). Twelve-to-twenty animals were tested for each drug at each concentration. Data represent means ± SEM of at least two independent assays.

To dive into the mechanism of action of caffeine, the effects of subtype-selective AR antagonists and agonists have been tested in *goa-1*[S47G] mutants. Specifically, antagonists were applied to assess their ability to mimic the effect of caffeine in ameliorating aberrant locomotor behavior, whereas agonists were challenged against caffeine to possibly counteract its beneficial effect. Our data showed that caffeine-induced reduction of the reversal rate is mimicked by the selective A_2A_R antagonists istradefylline, an FDA-approved molecule that is currently used in the treatment of PD (45), and ZM241385, as well as by the selective A_1_R antagonist DPCPX (Fig. 6A–C). Furthermore, the number of reversals per minute of wild-type nematodes was increased by the selective A_1_R agonist CPA (Fig. 6D), whereas the specific A_2A_R agonist CGS21680 led to inconsistent results (Fig. 6E). Of note, when challenged against caffeine, CPA counteracted the beneficial effect of this drug (Fig. 6F). Overall, these findings suggest that caffeine ameliorates the aberrant locomotor behavior of *goa-1* mutants acting, in part, through AR antagonism.

## Discussion

We report on the functional consequences of two *GNAO1* pathogenic variants associated with diverse clinical features. These variants, when inserted in the *C. elegans* genome, lead to hyperactive and uncoordinated locomotion, likely because of excessive release of acetylcholine by ventral cord motor neurons. Similarly, mutant animals show increased egg laying, suggesting lack of Gα_o_-mediated inhibition of neurotransmitter release by a different class of motor neurons (HSNs). Phenotypic profiling of homozygous and heterozygous animals establish that p.S47G and p.A221D behave as strong hypomorphic rather than complete LOF defects, with the latter exhibiting a partial DN effect. Our findings also reveal the potential role of caffeine and other AR antagonists in controlling *GNAO1*-related dyskinesia.

Since the first description of the disease (3), the number of patients with neurological disorders caused by *de novo GNAO1* mutations has rapidly grown up. According to ClinVar (https://www.ncbi.nlm.nih.gov/clinvar/), 60 pathogenic/likely pathogenic variants have been reported so far (53 missense, 5 in-frame deletions and 2 putative splice site changes; see (2) for the most up-to-date review). Missense variants affect 41 residues (Supplementary Material, Fig. S2), which are highly conserved among Gα_o_ orthologs (46). Worth noting, penetrance and expressivity of the main clinical features is largely dependent on the specific amino acid involved, with a few residues, including the mutational hot spot Arg^209^ and Glu^246^, which appear to be primarily associated with hypotonia and MD (1–3). However, a full understanding of the genotype–phenotype correlations as well as of the pathogenic mechanisms associated with individual mutations is still lacking.

In the original paper, Nakamura and colleagues (3) provided evidence indicating that localization of four Gα_o_ mutants (p.D174G, p.T191_F197del, p.G203R and p.I279N) to the plasma membrane and inhibition of calcium currents by norepinephrine was variably affected in heterologous cells, suggesting a LOF behavior of these variants on Gα_o_ signaling. Interestingly, mutations associated with both seizures and MD (p.T191_F197del and p.G203R) were characterized by a more severe LOF effect compared with variants underlying epilepsy without MD (p.D174G and p.I279N). More recently, a different scenario was proposed by Feng and coworkers (13) who pointed to aberrant cAMP production as the pathogenic mechanism of the disease. α_2A_ adrenergic receptor-mediated suppression of cAMP synthesis was evaluated in HEK293T cells transfected with pertussis toxin-insensitive Gα_o_ mutants. Based on the collected data, LOF (e.g. p.G40R, p.D174G and p.T191_F197del) and GOF (i.e. p.G42R, p.G203R and p.E246K) variants appeared to be primarily associated with EIEE17 and NEDIM, respectively. Several recurrent disease-causing variants, however, including those affecting the mutational hot spot Arg^209^, did not affect cAMP levels at all. Therefore, as correctly recognized by the authors, assessment of cAMP inhibition does not represent a fully informative readout for understanding the functional consequences of *GNAO1* mutations, at least in *in vitro* models or under the used experimental conditions. Finally, a recent paper by Muntean *et al.* (14) provided elegant evidence indicating that *GNAO1* variants disrupt signaling through dopamine and adenosine GPCRs in striatal neurons of mice by distinct molecular mechanisms. Besides their variable LOF behavior in signal transduction elicited by dopamine, only variants affecting residues located in the switch II and switch III regions (e.g. p.G203R, p.G204R, p.R209C, p.Q233P and p.E246K) were shown to behave as DN mutations interfering with the function of normal Gα_o_ via receptor trapping (p.G203R) or an alternative mechanism still to be defined (p.R209C). Conversely, variants involving residues located within or proximal to the P-loop (e.g. p.G42R, p.S47G and p.I56T) were LOF changes with no DN activity; rather these changes affect association with the βγ dimer. Of note, LOF and DN effects are not mutually exclusive: their relative degree of manifestation depends on the specific residue and substitution involved.

According to these data, our findings showed that *goa-1*[S47G] is a strong hypomorphic allele that does not act as a DN *in vivo*. Based on the localization of Ser^47^ in the P-loop motif, the LOF effect of the serine-to-glycine substitution is likely because of defective interaction with Mg^2+^ ions that, in turn, may lead to inefficient GTP binding. Interestingly, reduced affinity for Mg^2+^ has been described for other *GNAO1* variants affecting residues located far from the P-loop region, including Gly^203^. The LOF role of these changes was mainly attributed to a reduced ability of the switch II region to undergo the allosteric transition upon GTP binding needed to release the βγ dimer (47,48). Accordingly, this class of Gα_o_ mutants are deficient in both Gβγ release and signaling through GPCRs (14). Conversely, phenotypic analysis of *goa-1(+/A221D)* heterozygous animals revealed that p.A221D, which had not previously been functionally characterized in *in vitro* models, has a partial DN effect *in vivo*, at least on neurotransmitter release by HSN motor neurons mediating egg laying. Considering the possible effect of p.A221D on the conformation of the α5 helix, a key region mediating the interaction between the α-chain and the cognate receptor, the DN effect could be mediated by a more stable GPCR association, as previously reported for the p.G203R, p.G204R and p.Q233P mutants (14). Further studies are needed to untangle why the DN behavior of p.A221D was evident for the egg laying but not for the locomotor phenotype. Our data also showed that *goa-1* is a haploinsufficient gene for the particular phenotypes understudy here. Indeed, *goa-1(+/sa734)* null heterozygotes display a residual phenotype in terms of both unlaid eggs and reversal rate. This is in line with the observation that in the human population *GNAO1* is intolerant to LOF variation in the heterozygous state, according to gnomAD (pLI = 0.99). The reason why no frank LOF mutations including frameshift, stop-gain and out-of-frame splice site changes, have been reported so far in *GNAO1*-realated disorders remain elusive. These variants could be associated with a more severe phenotype causing embryonic lethality or, alternatively, to weaker clinical features. Mouse models do not help in this case. Although *Gnao1*^−/−^ mice show multiple neurological abnormalities including seizures and MD (11) and *Gnao1*^+/G203R^ (49) and *Gnao1*^+/R209H^ (50) knock-in mice phenocopy children harboring the corresponding mutations, heterozygous null mutants appear to be healthy (11). Further studies are warranted to unravel this conundrum.

No effective treatment is available for clinical conditions associated with *GNAO1* mutations. Concerning MD, tetrabenazine is the most used and effective drug, probably because of its widespread effect in reducing excitatory signaling through the depletion of biogenic amines. Other medications commonly used for the management of MD provided very modest efficacy. In patients with severely refractory MD, DBS has been reported to generally reduce the frequency and severity of MD exacerbations, although in the absence of complete remission or prevention of status dystonicus and further neurological deterioration (2). Based on these considerations, new therapeutic approaches are desirable for this devastating condition in order to prevent life-threatening dyskinetic status and the consequences of paroxysmal motor episodes on normal neuromotor development. Traditional preclinical drug discovery, however, is a complex and long-lasting process. Innovative approaches are needed to save time and resources. In this scenario, the use of *C. elegans* and other small invertebrates in the early phases of drug development has emerged as a promising strategy. Since its introduction as a model organism by Sidney Brenner in the early 70s (51), *C. elegans* has been widely used to discover and understand key biological processes, and to model human diseases (17). During the last decade, this nematode has also become a useful tool for low-to high-throughput screening of chemicals with potential therapeutic applications (52). Here, taking advantage of the easily recognizable phenotypes shown by *goa-1* mutants, we identified caffeine and other AR antagonists as putative drugs to control *GNAO1*-related hyperactive MD.

Caffeine is the most widely used psychoactive stimulant, known to influence sleep, cognition, learning and memory (53). In mammals, the beneficial role of this drug on higher cognitive functions and in delaying cognitive decline is principally mediated via non-selective inhibition of ARs (54–56). Specifically, the acute effect of oral caffeine intake was shown to occur via A_1_Rs and A_2A_Rs blockade, whereas chronic administration of caffeine modulates neuronal functions by inhibiting only A_2A_Rs (57). These GPCRs play a major role in the striatum and their blockade is neuroprotective in several disease models (58). Of note, caffeine is particularly suitable for chronic pharmacological treatments in humans since it can be administered orally and passes the blood–brain barrier. Accordingly, caffeine citrate is already available in the clinical setting for the treatment of primary *apneas* in premature newborns, highlighting its good safety profile for pediatric use (59). Worth mentioning, a clear relationship between increased caffeine consumption and decreased risk for PD has been established (60). Specifically, in PD models, A_2A_R ablation reduced L-DOPA-induced dyskinesia (LID) by modulating the effects of dopamine (61–64). Caffeine-induced improvement of cognitive performance in PD patients (65) further supports the view that this drug acts as a neuroprotective agent, with great benefits in terms of motor control (66). Finally, caffeine may improve motor symptoms in subjects with dominant *ADCY5* mutations (40,41), which cause a childhood-onset hyperkinetic MD reminiscent of NEDIM (12). A related clinical trial is currently in progress (ClinicalTrials.gov Identifier: NCT04469283).

In *C. elegans*, caffeine extends worm’s lifespan (67,68). This effect is reversed by concomitant exposure to adenosine, suggesting a conserved caffeine-AR pathway in the nematode (68). Of note, administration of 10 mm caffeine (the same concentration used in our experiments) to *C elegans* protects dopaminergic neurons from neurodegeneration elicited by excessive biosynthesis of endogenous L-DOPA (43), and improve aberrant locomotion induced by L-DOPA in a transgenic *C. elegans* model of PD (44). Specifically, Manalo *et al.* demonstrated that caffeine antagonism of a putative A_2A_R ortholog confers neuroprotection by increasing the availability of a dopamine D2-like receptor in a cAMP-independent manner (43). In this model, the protective role of caffeine is exerted by the synergistic action of adenosine and dopamine GPCRs, as suggested by the lack of efficacy of AR blockade without concomitant D2 receptor activation, and *vice versa*. This pattern evokes the interaction observed in higher vertebrate models, where A_2A_Rs modulate the indirect pathway of the basal ganglia, forming dimers with and antagonizing dopamine D_2_ receptors (69). A similar mechanism might explain the effect of caffeine as a bypass suppressor of aberrant locomotion observed in *goa-1* mutants. An AR-independent effect of caffeine, however, cannot be ruled out, particularly at the relatively high (mm) concentrations used in our assays. Indeed, caffeine may act as a non-specific phosphodiesterase inhibitor (70), promotes calcium release from intracellular stores (71) and inhibits phosphoinositide 3-kinases (72). Nevertheless, our pharmacological assays support a model in which the beneficial effect of caffeine on aberrant motor function of *goa-1* mutants is exerted, at least in part, via AR antagonism. To conclude, caffeine and istradefylline have emerged as candidate pharmacological treatments for *GNAO1*-related MD; the potential clinical suitability of these drugs is strongly supported by their well-characterized safety, being already available for the treatment of primary *apneas* in premature newborns (59) and PD (45), respectively.

Summarizing, this work establishes a *C. elegans* model of neurodevelopmental disorders caused by *GNAO1* mutations. The powerful toolkit of *C. elegans* genetics may help to disclose the mechanisms of action and to provide a significant boost to drug discovery. The current lack of effective therapies and the severity of the disease with life-threatening events strongly encourage to use this and other simple model organisms as a platform for genetic and drug screening.

## Materials and Methods

### Clinical and structural findings

Written informed consent to disclose clinical information was obtained from the parents of the index patient harboring the c.139A > G (p.S47G) variant according to the local institutional policy. Structural modelling was developed by using the UCSF Chimera software (73).

### C. elegans studies

Culture, maintenance, injections and genetic crosses were performed using standard techniques (74,75). The Bristol N2 (control animals), DG1856 *goa-1*(*sa734*) and COP1863 *goa-1*(*knu751*) strains were provided by the CGC (University of Minnesota, Minneapolis, MN).

The engineered change to the endogenous *goa-1 locus* was carried out by CRISPR/Cas9, as previously described by the authors (76,77). Twenty wild-type animals have been injected with a mix containing 750 ng/μl Cas9 (IDT, Coralville, Iowa, USA), 700 ng/μl ALT-R CRISPR tracrRNA (IDT), 115 ng/μl *dpy-10* crRNA (5′-CTCGTGGTGCCTATGGTAGC-3′), 37.5 ng/μl ssODN *dpy-10* (5′-ATAGGCTGTTGGTCTGAAGCCATGTGAAGCTCCGCTACCATAGGCACCG CATGCGGTACGGTTTCCAGTCATTCTCATCTTGCCGTATTGAAGTTCAAGTG-3′), 350 ng/μl *goa-1* crRNA (5′-AAACTGCTGCTACTTGGTGC-3′) and 175 ng/μl ssODN *goa-1*[S47G] (5′-TTAAAGAAGACGGCATGCAAGCGGCAAAAGATATCAAGCTTCTCCTTCTCGGAGCTGGTGAGTCTGGTAAGGGAACAATCGTAAAACAGATGAAGTGAGATTTTTTTAAATTTCT-3′). Injected worms were recovered on nematode growth medium (NGM) at 20°C, each in a separate plate. Animals with a roller (rol; indicating the insertion of a heterozygous, disrupting mutation in the *dpy-10* gene) or a dumpy (dpy; indicating the insertion of a homozygous, disrupting mutation in the *dpy-10* gene) phenotype were isolated, as well as pools of five wild-type hermaphrodites from jackpot plates (i.e. plates with several rol and dpy animals). To isolate worms harboring the *goa-1* c.139_141TCG > GGA (p.S47G) nucleotide change, PCR amplification was performed using a single forward primer (5′- GAGGATATCAAGTGGAGACC -3′) and two reverse primers annealing with the wild-type (5′- CGTGTTACTGTAGACAACC -3′) or the modified (5′- CCTTACCAGACTCACCAGCT -3′) sequence. Homozygosity was confirmed by Sanger sequencing using standard techniques. Two independent lines holding the desired change were generated; both were out-crossed twice to the N2 strain to remove any potential off-target mutation and were then used for further analyses and crosses. These two lines showed an equivalent phenotype and were designated as *goa-1*(*pan5*[S47G]).

Sensitivity to aldicarb, levamisole and PTZ (Sigma-Aldrich, Saint Louis, MO) was determined as previously described (78–81). Stock solutions were prepared according to manufacturer’s instructions. In all assays, young adults obtained from synchronized cultures were tested. Paralysis was confirmed by assessing lack of response to prodding with a platinum wire or an eyebrow hair and absence of pharyngeal pumping. Convulsions were assessed on agar plates and/or liquid solution. Phenotypic analyses were conducted at a Leica MZ10F and a Nikon SMZ18 dissecting microscopes. Data were analyzed as the percentage of animals that still moved at each time-point. Because of day-to-day variability, comparisons were made up only between worms assessed on the same day and with the same batch of drug. Caffeine (1,3,7-trimethylxanthine; Sigma Aldrich) was freshly dissolved in water and added into just-agar plates (5 mm Potassium phosphate buffer pH 6.0, 1 mm CaCl_2_, 1 mm MgSO_4_) to a final concentration of 1, 10 or 20 mm. Plates were prepared the day before use, seeded with 12.5 μl of *E. coli* OP50 bacteria to obtain a thin and homogeneous lawn of food. The reversal rate was assessed in L3 animals moved on the assay plate following exposure to caffeine (1–4 h or overnight). To assess the effect of chronic exposure, young adults were moved to the assay plate to let them lay eggs, and the number of reversals per minute was assessed in the F1 progeny at the L3 stage. Sensitivity to subtype-selective ARs agonists/antagonists (Tocris) was assessed as described previously after 2 h exposition. DPCPX (8-Cyclopentyl-1,3-dipropylxanthine) was freshly dissolved in ethanol. Istradefylline (8-[(1E)-2-(2-(3,4-Dimethoxyphenyl)ethenyl]-1,3-diethyl-3,7-dihydro-7-methyl-1H-purine-2,6-dione), ZM241385 (4-(2-[7-Amino-2-(2-furyl)[1,2,4]triazolo[2,3-a][1,3,5]triazin-5-ylamino]ethyl)phenol), CPA (*N*-cyclopentyladenosine) and CGS21680 (4-[2-[[6-Amino-9-(*N*-ethyl-β-d-ribofuranuronamidosyl)-9H-purin-2-yl]amino]ethyl]benzenepropanoic acid hydrochloride) were freshly dissolved in DMSO. Since ethanol and DMSO were shown to affect the reversal rate in *goa-1*[S47G] (0.2 and 1% ethanol increases the reversal rate of 1.8 and 3.6%, respectively; 0.2 and 1% DMSO decreases the reversal rate of 3.1 and 8.9%, respectively) and N2 animals (0.2 and 1% DMSO decreases the reversal rate of 38 and 50%, respectively; 0.2 and 1% ethanol increases the reversal rate of 8 and 10%, respectively), worms were treated in parallel with the drug or the solvent at the corresponding concentration. The CPA/caffeine competition assay was performed by adding both drugs into just-agar plates (caffeine, 10 mm; CPA, 20 or 100 μm). The percentage of rescue is defined as [1 − (Δ1/Δ2)∙100], where Δ1 and Δ2 represent the difference between the number of reversals per minute in the presence and absence of the drug, respectively, and the number of reversals per minute of wild-type nematodes treated with the solvent at the corresponding concentration. In other words, the percentage of rescue is 0% when the reversal rate remains the same before and after drug exposure and 100% when the reversal rate following drug exposure corresponds to that of wild-type animals.

Locomotion was analyzed by crawling assay at a Nikon SMZ18 stereomicroscope (82). To measure the locomotor behavior quantitatively, groups of *C. elegans* have been recorded using an *in-house* automated tracking system under uniform and steady illumination, on 35 mm petri dishes spread with a thin lawn of *E. coli* OP50 bacteria, with a greyscale camera (Mako U-130 camera, Allied Vision GmbH, Stadtroda, Germany), at 3 frames per second for 10 min. The petri dishes were placed on a transparent plexiglass panel. The light illuminates the bottom of the petri dish and is emitted by a matrix of white light LEDs (12 W Panel SMD LED Ceiling Down Light Bulb Lamp) under the plexiglass panel. The camera is placed at a calibrated height over the petri dish and collects the light passing through the sample with a zoom lens with 18/108 range and F2.5 that conveys the light on the camera sensor (1280 × 1024 pixels). A filter film (Gold privacy filter, 3 M) is placed between the plexiglass and the petri dish to increase contrast and image quality. Videos were post-processed with a custom-made MATLAB software (Mathworks, Natick, Massachusetts) through a gaussian filter and then thresholded. *Caenorhabditis elegans* were detected based on the size of the objects in the thresholded image. Too large or too small objects were discarded. Trajectories were created based on the proximity of the centroid (corresponding to the mean position of the points belonging to the object) in all consecutive frames, and then validated visually one by one. Velocity was calculated based on the distance between the centroids of objects of the same trajectory in consecutive frames, whereas reversals were defined as portions of the trajectory that present a bend with an angle greater than 50°, as described in (83). The fraction of time of single tracks spent in a coiled position was calculated based on the presence of holes in the tracked object and its velocity. Body bends were defined as events in which the mid-body point of the nematode reaches the highest curvature in the opposite direction from the previously counted bend, while moving.

Neurotransmitter release by HSN motor neurons was explored by counting the number of eggs in the uterus of adult hermaphrodites. Adult animals of each genotype were examined on OP50-seeded NGM plates during the exponential phase of laying, and the number of eggs retained in the uterus of hermaphrodites has been counted using an Eclipse Ti2-E microscope (Nikon Europe, Florence, Italy) equipped with DIC optics on live animals mounted on 2% agarose pads containing 10 mm sodium azide as anesthetic. The number of laid eggs was also investigated. Fresh NGM plates were seeded with 25 μl of OP50 the day before the experiment and bacteria were allowed to grow at RT overnight. Afterwards, twelve L4 hermaphrodites per each genotype were cloned in the assay plates. Subsequently, every 24 h until the end of the laying period, adult animals were transferred to new assay plates and the number of eggs, larvae, and adults were counted in each assay plate.

Genotype blinding was used for all experiments except for the acquisition of microphotographs shown in Figure 1.

### Statistics

Significant differences were calculated using the GraphPad Prism 8.4.2 software. To compare the speed distributions associated with different strains we performed the two-sample *t*-test at the 5% significance level with MATLAB (Mathworks, Natick, Massachusetts).

## Supplementary Material

Supplementary_Material_ddab296
