# Supplementary material for: Caenorhabditis elegans provides an efficient drug screening platform for GNAO1-related disorders and highlights the potential role of caffeine in controlling dyskinesia
Source: Hum Mol Genet. 2021 Oct 8;31(6):929–41. doi: 10.1093/hmg/ddab296 (PMC8947233; doi:10.1093/hmg/ddab296)
Supplement: Supplementary_Material_ddab296 [file supplementary_material_ddab296.docx]

**Supplementary Material**


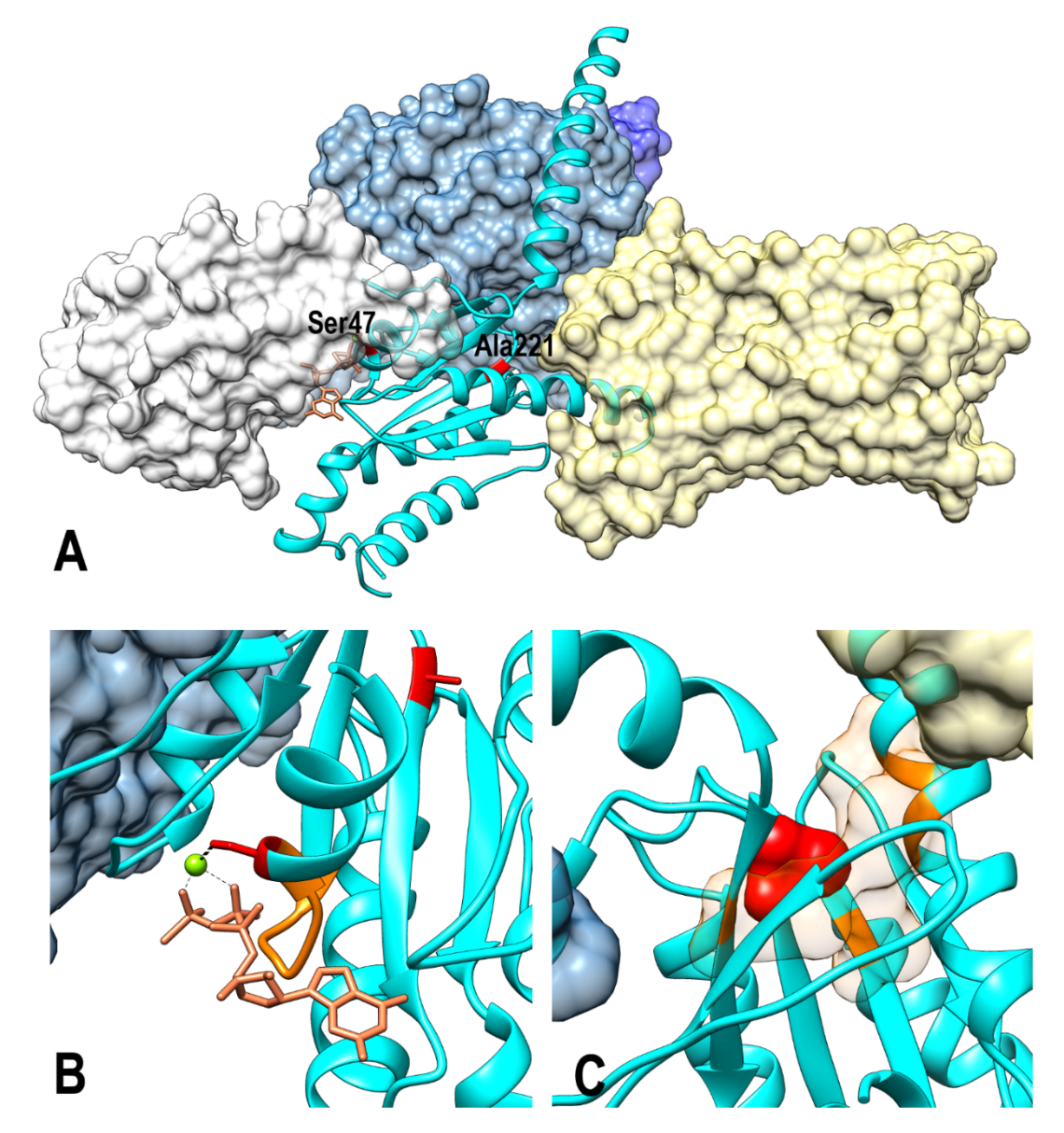


**Figure S1**. **Structural features of the Ser^47^ and Ala^221^ residues**. Structure of the complex between the heterotrimeric G_o_ protein and the serotonin 5-HT1B receptor (pdb code 6g79). (**A**) In the heterotrimeric G_o_ protein, the RAS-like GTPase domain of the α-subunit is reported as cyan ribbon, whilst the β- and γ-subunits are reported as semi-transparent light-blue and blue surfaces, respectively. The serotonin 5-HT1B receptor is reported as semi-transparent light yellow surface. The ribbon of residues Ser^47^ and Ala^221^ is coloured in red and their side-chains are reported as red sticks. The white semi-transparent surface represents the helical domain of the α-subunit in the heterotrimeric protein. The helical domain was lacking in the 6g79 structure and it has been taken for similarity from the structure of the G_i_ α1 protein (pdb code 1gil), after superimposition of the RAS-like GTPase domains. Similarly, the analogue of the GTP, 5'-guanosine-diphosphate-monothiophosphate (GSP), and the Mg atom, absent in 6g79 structure, were added from 1gil and reported as coral sticks and green sphere, respectively. (**B**) Enlargement of the region comprising the Ser^47^ residue. Ser^47^ is part of the P-loop (in orange in this panel). The interactions stabilizing the Mg atom, including that involving the hydroxyl group in Ser^47^, are reported as dashed black lines. (**C**) Enlargement of the region surrounding the Ala^221^ residue. In this, panel Ala^221^ is reported as red surface. The ribbon of the residues belonging to the hydrophobic core (Ala^221^, Leu^36^, Ile^266^, Val^339^, and Ile^342^) is coloured in orange and the residues are reported as semi-transparent yellow surface.


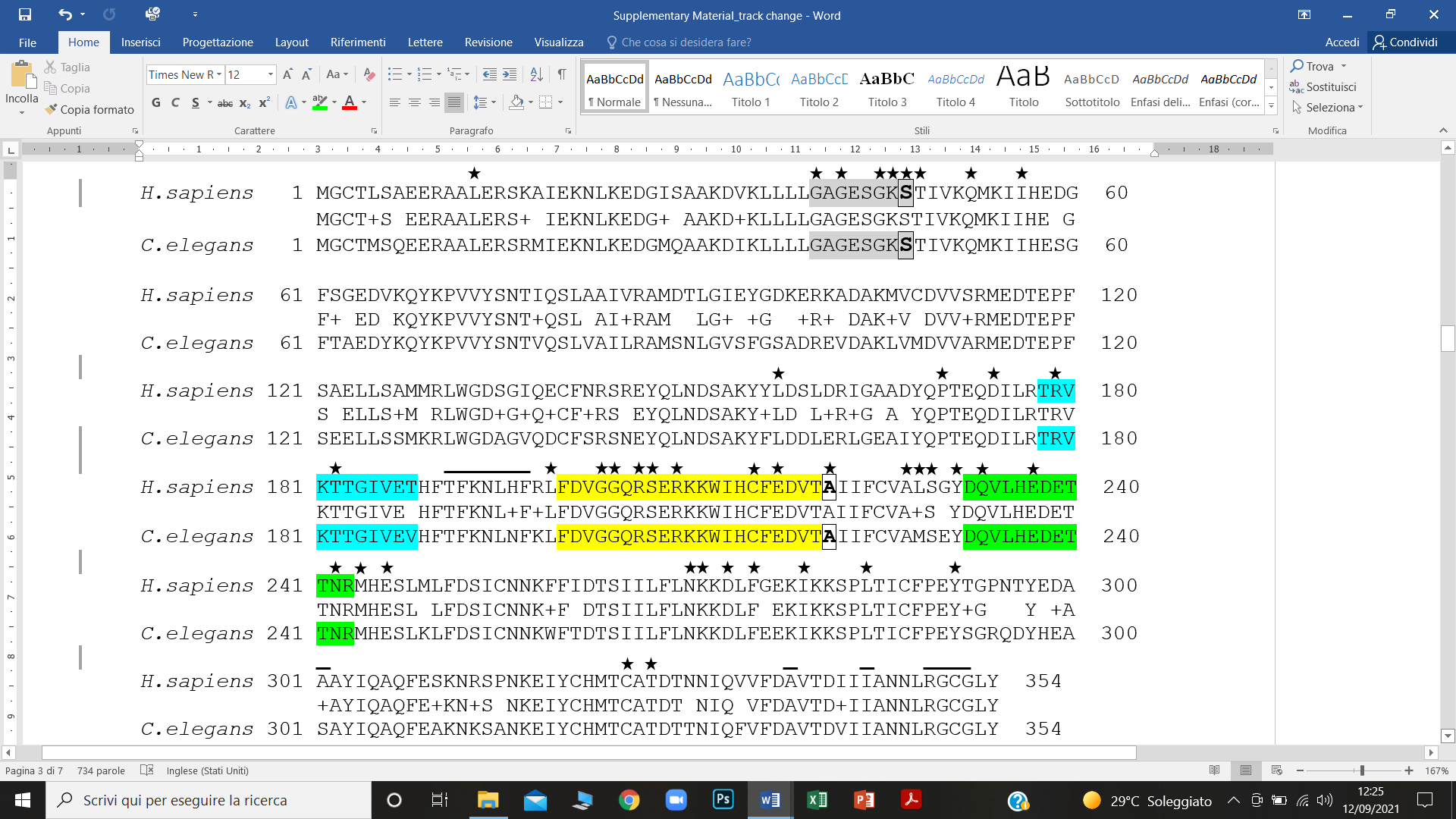


**Figure S2.** **Amino acid sequence alignment of human GNAO1 and the *C. elegans* GOA-1 ortholog** (<https://www.ncbi.nlm.nih.gov/homologene>). Black stars indicate affected residues in *GNAO1*-related disorders (<https://www.ncbi.nlm.nih.gov/clinvar/>). Solid lines indicate in-frame deletions/delins. Rare splice site variants (c.723+1G>A and c.723+2T>A) are not reported. The mutated residues studied in this work are highlighted in bold and included in a box. The P-loop consensus motif and the switch I, switch II, and switch III regions are highlighted in gray, cyan, yellow and green, respectively (ref. 30). Identity and conservation (+) of individual residues is also reported (middle row).

**
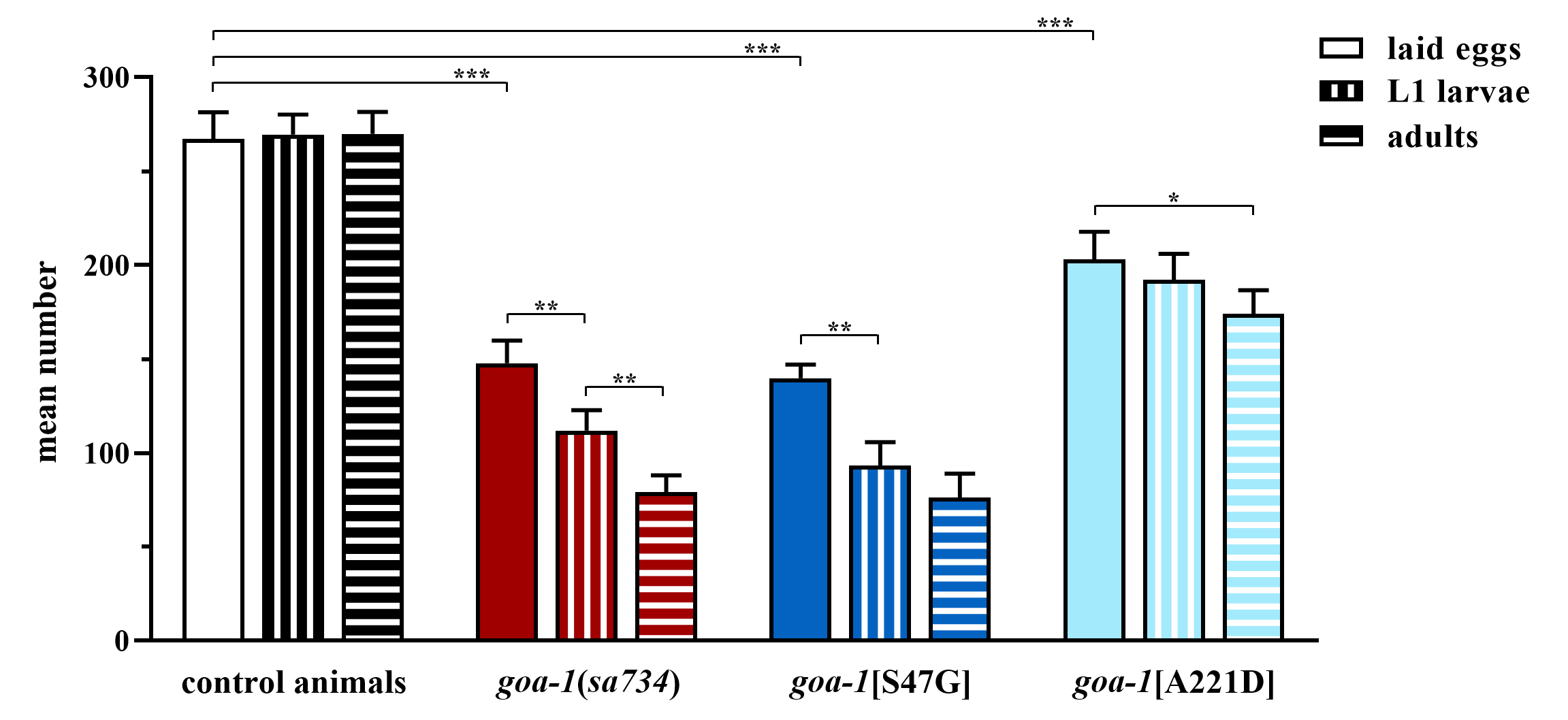
**

**Figure S3. Brood size and developmental programs are affected by *goa-1* mutations.** *goa-1*(*sa734*) null mutants and both *goa-1*[S47G] and *goa-1*[A221D] knock-in animals show a significantly reduced brood size compared to control worms (****p*<0.0001 in all comparisons; two-way ANOVA with Bonferroni correction) and exhibit low penetrant embryonic and/or larval lethality (**p*<0.05 and ***p*<0.01). Twenty hermaphrodites per genotype were assayed. Data represent means ± SEM.

**
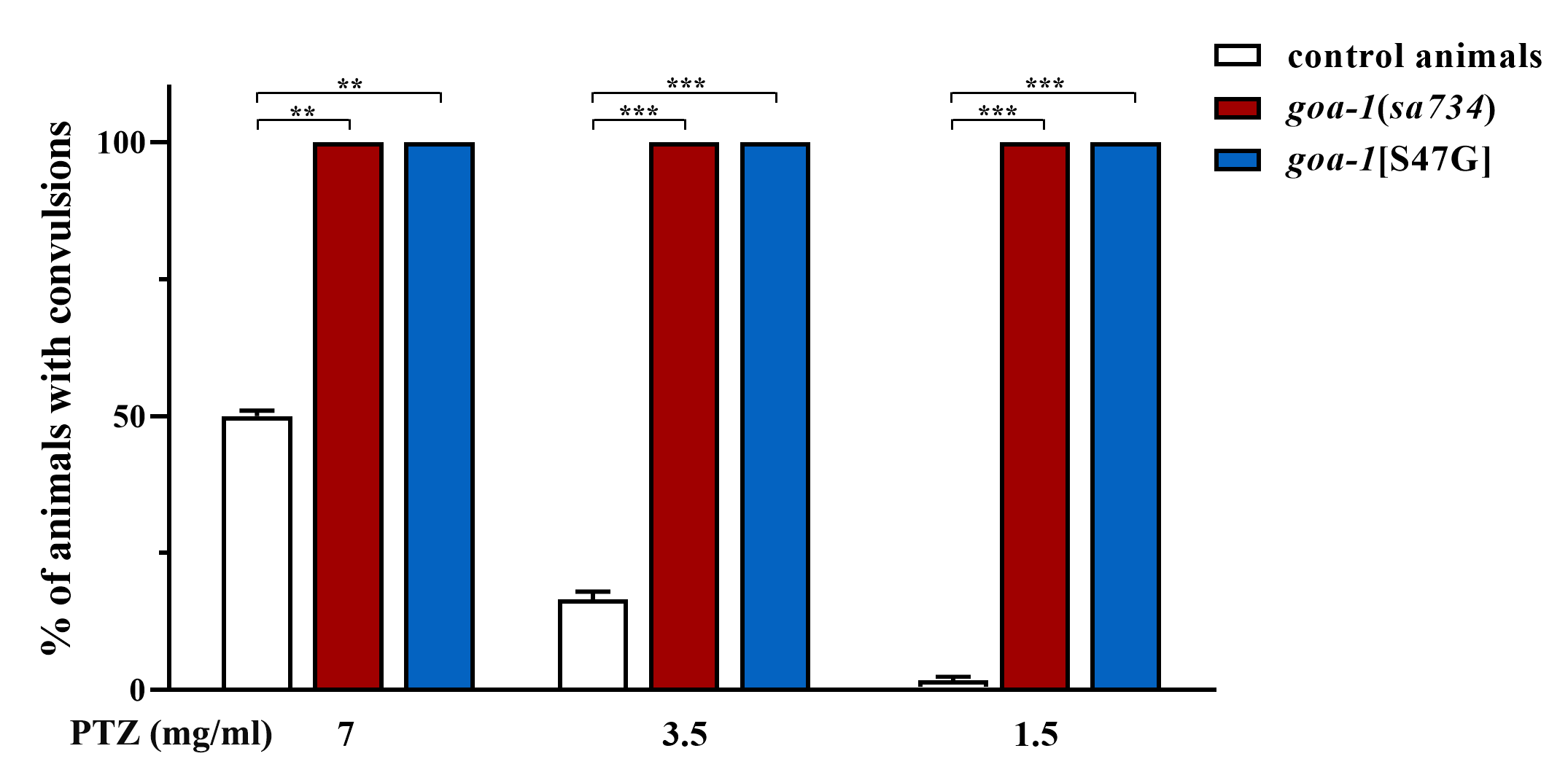
**

**Figure S4. PTZ assay in liquid solution.** *goa-1*(*sa734*) null mutants and *goa-1*[S47G] animals display hypersensitivity to PTZ-induced convulsions. This assay was performed in liquid solution using the indicated drug concentrations, and confirmed data obtained on agar plates (***p*<0.005 and ****p*<0.0001; two-way ANOVA with Bonferroni correction). Fifteen animals for each genotype and experimental condition were tested. Data represent means ± SEM of three independent experiments. *goa-1*[A221D] animals were not tested in liquid solution.


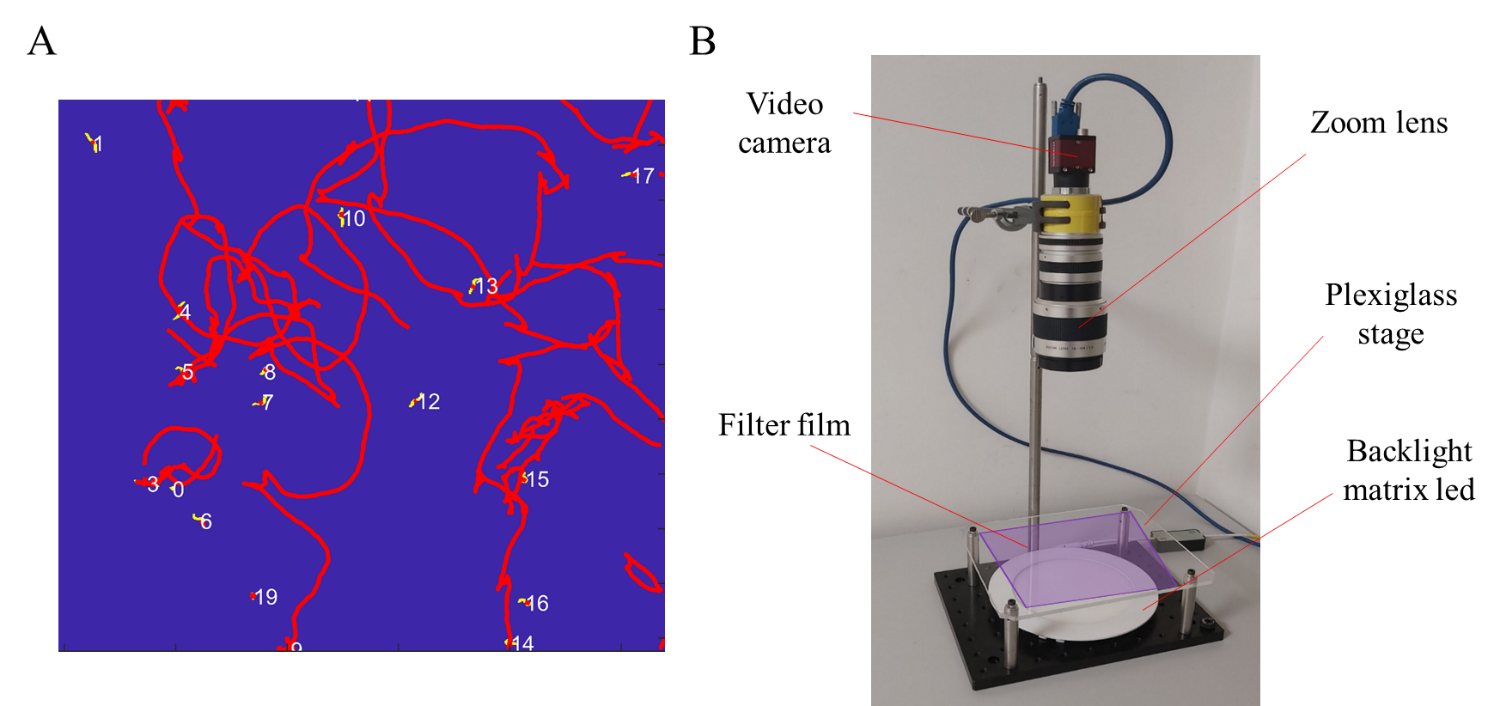


**Figure S5. Automated tracking system setup.** (**A**) Representative traces recorded on a 35 mm petri dish seeded with a thin lawn of *E. coli* OP50 bacteria. Numbered yellow objects represent *C. elegans* outlines, while red tracks represent their trajectories. (**B**) Experimental setup for behavioral assays: video camera and zoom lens are supported by a metallic structure with an adaptable height. The zoom lens is upside down and captures the top of the plate, which is placed on a plexiglass support. To increase the quality of the image, a light filter film is positioned on the plexiglass. The illumination system comprises a white matrix LED in background configuration, which is located on the metallic base, under the plexiglass support.


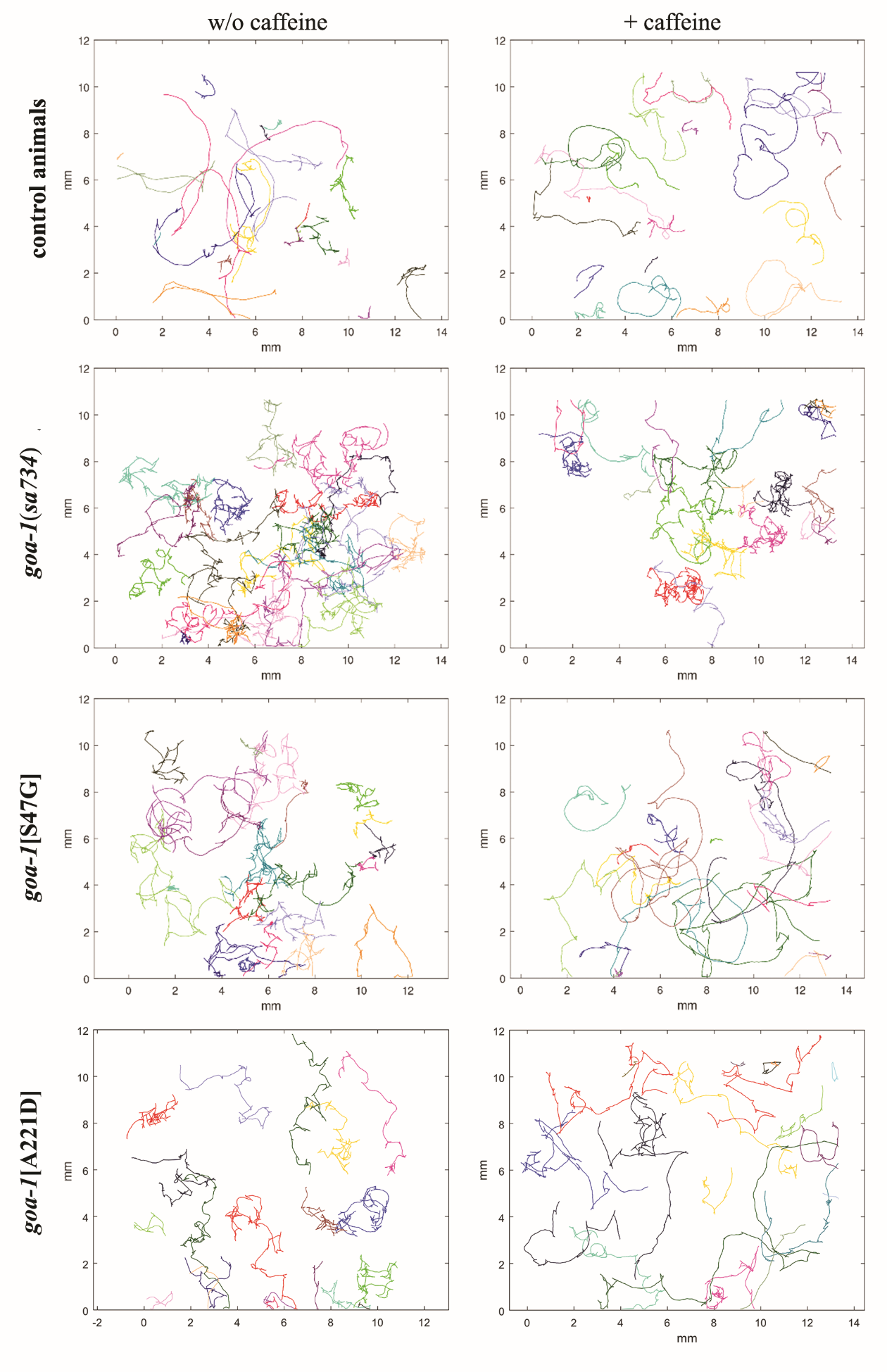


**Figure S6. Computational analysis of *C. elegans* trajectories following exposure to caffeine.** Trajectories of multiple *C. elegans* (n=20) on 35 mm plates seeded with a thin lawn of *E.coli* OP50 bacteria with or without 10 mM caffeine. Animals were recorded for 10 minutes. Different colors refer to different nematodes. All mutants show hyperactive crawling, with a higher frequency of reversals, compared to controls. Exposure to caffeine rescues, in part, these phenotypes.
